# Ultrasound and magnetic resonance imaging of fetal gastrointestinal tract disorders

**DOI:** 10.1007/s00261-025-05330-y

**Published:** 2025-12-19

**Authors:** Leonor Alamo, Carole Gengler, Joanna Sichitiu

**Affiliations:** 1 University Hospital of Lausanne, Lausanne, Switzerland; 2 University of Lausanne, Lausanne, Switzerland

**Keywords:** Prenatal diagnosis, Gastrointestinal pathology, screening ultrasound, Prenatal magnetic resonance imaging

## Abstract

This article describes the prenatal imaging findings on ultrasound and magnetic resonance examinations of the main congenital anomalies and complications affecting the fetal digestive tract. The findings of the two methods are compared and correlated, the indications for performing complementary magnetic resonance imaging are discussed, and the relevance of the information provided by this technique is evaluated.

## Introduction

Routine prenatal ultrasound (US) screening has improved the detection of fetal gastrointestinal disorders. The most common congenital digestive anomaly identified *in utero* is uni- or multifocal atresia, which is often associated with severe, persistent polyhydramnios and causes fluid accumulation in the digestive tract proximal to the level of the obstruction and distal collapse [1–4]. Enteric duplication cysts are the most frequently observed benign digestive fetal masses. They may arise from any part of the gastrointestinal tract and may communicate with the intestinal lumen [5, 6]. Fetal digestive complications include aseptic meconium peritonitis after intestinal infection or perforation and the rarely detected *in utero* midgut volvulus [7, 8].

Screening US is the gold standard imaging method for prenatal diagnosis. However, magnetic resonance imaging (MRI) is progressively expanding its indications as a complementary method, proving itself especially useful for confirmation of suspected malformations and for providing further information in case of incomplete or inconclusive ultrasonographic findings [9–11]. Although T2-weighted (T2-W) MRI sequences are the basis for prenatal diagnosis, multiplanar T1-weighted (T1-W) sequences also provide crucial information in cases of digestive pathology, because the different chemical composition and signal characteristics of the fluids filling the proximal and distal gastrointestinal tract in T1- and T2-W sequences facilitate accurate location of the pathology, thereby reducing the differential diagnosis [4, 12, 13].

This pictorial essay demonstrates the advantages of performing complementary MRI examinations in selected fetuses with gastrointestinal malformations or complications detected on US. It discusses the main indications for performing MRI, compares and correlates US and MRI imaging findings, and analyzes the relevance of additional information provided by this method. The images and clinical data are from patients who were diagnosed and treated at our hospital, which is a tertiary reference center for fetal diagnosis.

## Prenatal magnetic resonance imaging

Fetal MRI is always a complementary imaging method that should only be performed after the detection of abnormal or inconclusive findings on a prenatal US performed by experienced physicians. The main indications for the gastrointestinal tract are the identification of the level of a suspected obstruction, the anatomic evaluation and detection of unexpected malformations in fetuses with syndromes or complex pathologies, and the evaluation of digestive complications *in utero* before taking decisions about further pregnancy management [14].

The optimal time to perform a fetal MRI is the third trimester of pregnancy, between the 26 and 32 gestational weeks (GW), because most fetal organs can be visualized in detail at this time [15]. The standard prenatal protocol should include T1- and T2-W fast-spin-echo sequences in at least three fetal spatial planes. Multiplanar T1-W sequences evaluate the distribution, quantity, and signal characteristics of the fluids filling the gastrointestinal tract whereas T2-W sequences allow accurate evaluation of the fetal anatomy [4, 12, 13].

## Normal fetal gastrointestinal tract on US and MRI

**Fig. 1:**
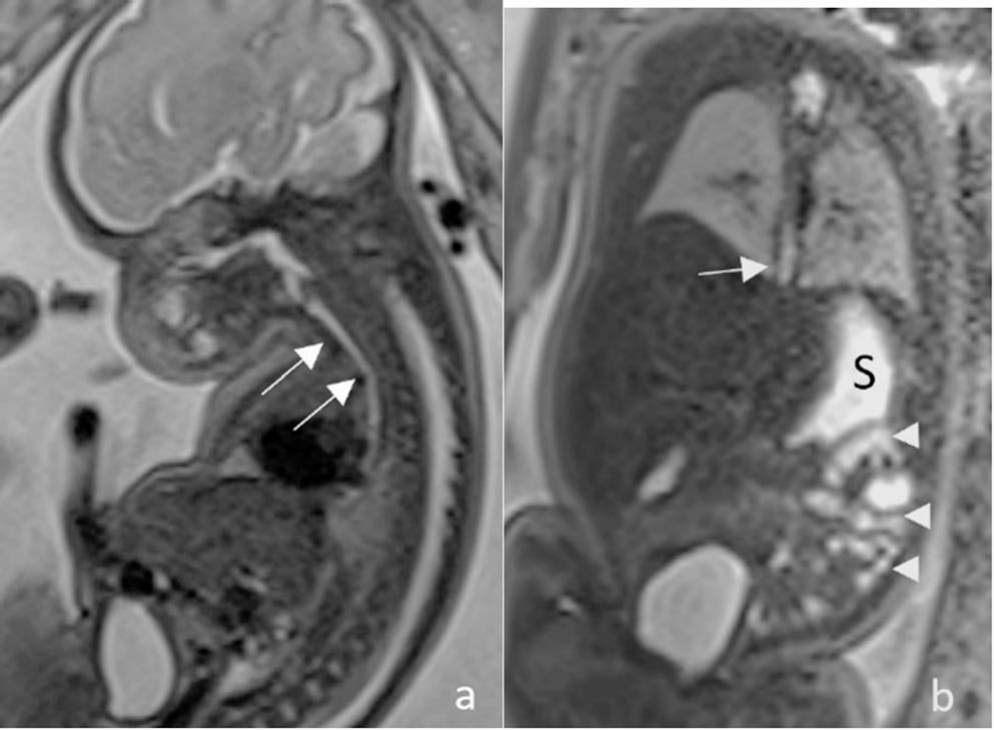
Normal morphology of the proximal digestive tract Sagittal (**a**) and coronal (**b**) T2-W MR images at 34 gestational weeks (GW) show the esophagus as a fluid-filled, regular thin tubular structure in the posterior mediastinum (arrows, **a**-**b**). Fluid filling the esophagus, stomach (S), and proximal small bowel has the same signal intensity as the amniotic fluid. Note the homogeneous diameter of the proximal bowel loops (head arrows, **b**)

**Fig. 2:**
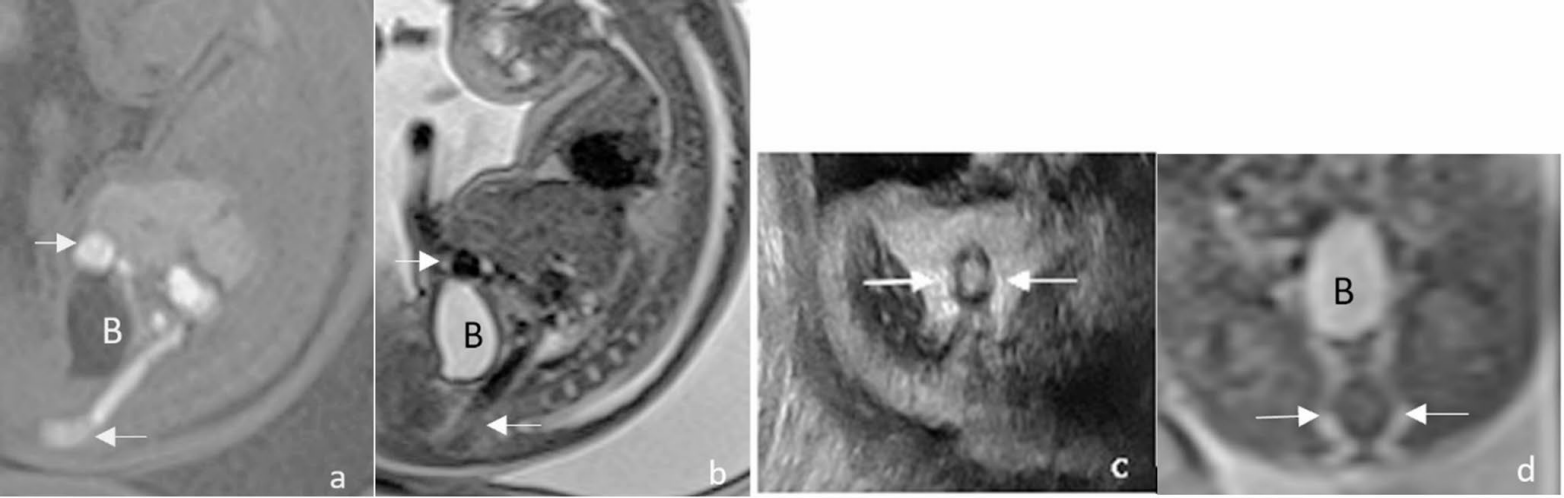
Normal morphology of the distal digestive tract Midline sagittal T1- (**a**) and T2-W (**b**) MR images at 30GW show normal meconium filling the colon and rectum (arrows), homogeneously hyperintense on T1- and hypointense on T2-W images. US (**c**) and axial T2-W MR (**d**) images of the fetal perineum at 32GW show the normal anal complex (arrows) with central located “target sign”. B: Bladder

In a normal fetus, the esophagus can be observed as a fluid-filled, regular thin tubular structure in the posterior mediastinum (Fig. 1). However, it is often collapsed and is therefore not always identifiable. Continuous fetal swallowing should ensure that the stomach, duodenum, and proximal small bowel are always filled with fluid that has identical characteristics to the amniotic fluid, being anechoic on US and homogeneously hypointense on T1-W and hyperintense on T2-W MR sequences [12, 13]. The diameter of the proximal small bowel loops in the left hemiabdomen should be homogeneous and not exceed 7 mm (Fig. 1). The chemical composition and signal characteristics of swallowed amniotic fluid change during its passage through the enteric tract due to fluid reabsorption, secretion by the liver and intestinal glands, and intestinal epithelium desquamation [4]. The resulting meconium fills the colon and rectum and is rich in proteins, being homogeneously hyperintense on T1-W and hypointense on T2-W sequences (Fig. 2) [2, 4, 16, 17]. Indeed, one of the advantages of MRI over US in the examination of the gastrointestinal tract is MRI’s ability to identify meconium [18].

The size of the colon increases with gestational age but rarely exceeds 20 mm in diameter and should always remain smaller than the bladder diameter. A gradual increase in the anal sphincter pressure after the 20 GW leads to meconium retention, which progressive accumulation from the distal to the proximal segments of the intestinal tract. In a normal fetus, meconium will be only visible in the rectum at the 20GW, in the whole colon at the 24GW, and in the distal ileal loops at the end of pregnancy (Fig. 2) [16, 17]. Rectal meconium should extend > 1 cm below the neck of the bladder in midline sagittal images [16]. The anal complex is easier to recognize on US than on MRI and can be identified in 90–100% of cases at screening US examinations performed at the 23–24 GW [19]. MRI often requires a T2-plane oriented to the fetal perineum. In this plane, the anus appears as a midline structure with a characteristic “target”-like form, composed of the central hyperintense mucosa surrounded by the hypointense sphincteric rim [2, 3] (Fig. 2).

## Gastrointestinal congenital pathology

### Isolated gastrointestinal atresia

The most frequently detected congenital digestive anomaly is isolated atresia [1, 4]. Usually, the first detected ultrasonographic findings are persistent polyhydramnios and dilatation of the digestive tract located proximal to the obstruction [5].

**Fig. 3:**
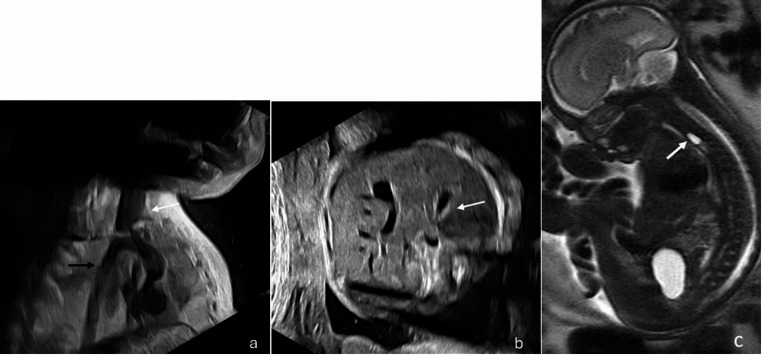
Prenatal imaging findings of esophageal atresia (EA) Case 1 (**a**, **b**) EA with associated TEF: sagittal midline cervical plane (**a**) shows a distended proximal esophagus (white arrow, **a**). The black arrow (**a**) shows the thoracic aorta. Transverse US image of the upper abdomen (**b**) reveals an anomalous small stomach (arrow, b). Case 2 (**c**), pure EA. Midline sagittal T2-W image MR at 32GW reveals the esophageal pouch in the posterior mediastinum (arrow, **c**), filled with homogeneous hyperintense fluid, and absent stomach

Esophageal atresia (EA) mostly occurs at the level of tracheal bifurcation. It is the most common type of digestive obstruction, accounting for approximately 35% of all cases, with a reported incidence of 1:2000–3000 live births. It appears as an isolated anomaly in approximately 60% of patients and as part of a syndrome or associated with other malformations in the remaining 40% [19, 20]. Prenatal US diagnosis accounts for 22%−31.7% of all cases [20–22]. Suggestive imaging findings include severe and persistent polyhydramnios, detected in approximately 56% of cases, variable distension of the hypopharynx, a dilated proximal esophagus or “cervical pouch sign”, and a persistent absent or small-sized stomach (50%) [13, 23] (Fig. 3). When performed following a suspicious US, MRI has good diagnostic accuracy, with a sensitivity of 94.7% and a specificity of 89.3% [22]. Midline sagittal dynamic cine-mode T2-W sequences during fetal swallowing may improve cervical pouch visualization and predict gap length [24].

In approximately 85% of patients with EA there is an associated tracheo-esophageal fistula (TEF) [20, 22]. Its antenatal diagnosis remains challenging, as a distal fistula can allow passage of fluid and modify the previously described imaging findings, rendering them absent or less evident. In patients with TEF, the amniotic fluid may be normal, and both the esophageal pouch and distended hypopharynx are less likely to be observed. The size of the stomach and the visualization of the distal esophagus are the key findings indicative of this diagnosis. However, although fetal MRI helps to improve diagnostic accuracy, the rate of prenatal detection remains low [22, 25] (Fig. 3).

**Fig. 4:**
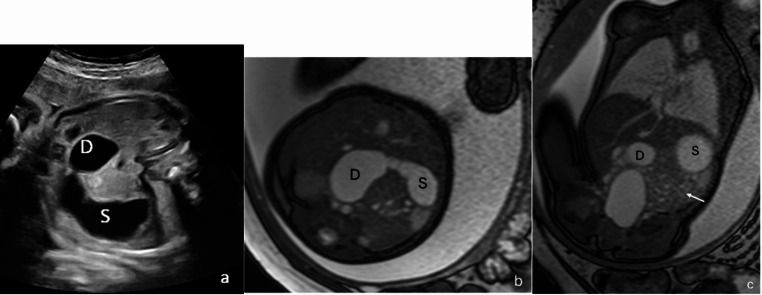
Prenatal imaging findings of duodenal atresia Transverse (**a**) screening US image at 23GW and transverse (**b**) and coronal (**c**) T2-W MR images at 30 GW show the classic “double bubble sign” with distended stomach (S) and proximal duodenum (D). Note increased amniotic fluid quantity in (**b**) and collapsed left bowel loops (arrow, **c**)

The incidence of duodenal atresia ranges from 1–3:10000 live births, with approximately 60% comprising of isolated cases, and 40% associated with other congenital anomalies. These are mainly cardiac, genito-urinary, and chromosomal, like Down’s syndrome [17]. The European registry of congenital anomalies working group (EUROCAT) reports a prenatal US detection rate of 52–54% [20, 21]. Suggestive imaging findings include severe, persistent polyhydramnios and the classic “double bubble sign”, which graphically describes the distension of the fluid-filled stomach and the proximal duodenum (Fig. 4). However, this sign may be a false-positive and represent a transient finding in an otherwise healthy fetus [26]. In the case of inconclusive ultrasonographic findings, MRI can confirm the diagnosis and help to identify concomitant congenital anomalies, including multiple levels of atresia.

**Fig. 5:**
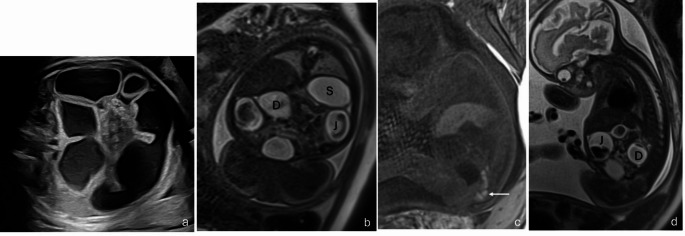
Imaging findings of proximal small bowel atresia with secondary volvulus. Transverse image (**a**) of screening US at 25GW, coronal T2- (**b**) and sagittal T1- (**c**) and T2-W (**d**) MR images at 30GW show distended stomach (S), duodenum (D) and proximal jejunal loops (J), filled with fluid isointense to the amniotic fluid. Note collapsed distal bowel loops, but normal-sized rectum, and normal intensity of distal meconium (arrow, **c**). Pregnancy was interrupted due to increased bowel dilatation and reduced peristalsis

Small intestine atresia (jejunal or ileal) has an incidence range of 1:3000–5000 births and represents approximately 32% of all atresia [20]. Associated anomalies, also largely gastrointestinal, are observed in approximately 23% of cases [4, 22, 27]. The main imaging findings include persistent polyhydramnios and fluid-filled distension of the bowel loops located proximal to the atretic segment (Fig. 5) [28–31]. The prenatal detection rate is highly variable, ranging from 25 to 90% [4, 20]. Real-time ultrasonographic evaluation may identify hyperperistalsis of the distended bowel proximal to the obstruction, but accurate identification of the level and length of the obstruction is not always possible [31, 32]. Indeed, one of the main indications for MRI is the identification of the location of the atresia based on the number of dilated loops, the signal intensity characteristics of the fluid filling these loops, the size of colon and rectum, and the quantity and signal characteristics of meconium. These findings become more evident in the third trimester of pregnancy.

A proximal jejunal atresia shows homogenous T1-hypointense and T2-hyperintense fluid in the dilated bowel, and an almost normal-sized colon and rectum, filled with normal meconium, produced by the intestinal secretions distal to the atresia (Fig. 5). A mid-small bowel obstruction displays a higher number of distended loops, filled with intermediate T1- and T2-W signal, and delayed detection and reduced quantity of distal meconium. Finally, a distal ileal atresia features multiple dilated loops, some of which are filled with meconium-like fluid signal, and delayed detection as well as scarce quantity of colorectal meconium. Reduced colon and rectum diameter or “microcolon” are also associated [9, 33]. An isolated jejunal or ileal atresia has an excellent long-term outcome after neonatal repair, but its association with the most important differential diagnosis - cystic fibrosis- considerably increases the risk of fetal morbidity and mortality [4].

**Fig. 6:**
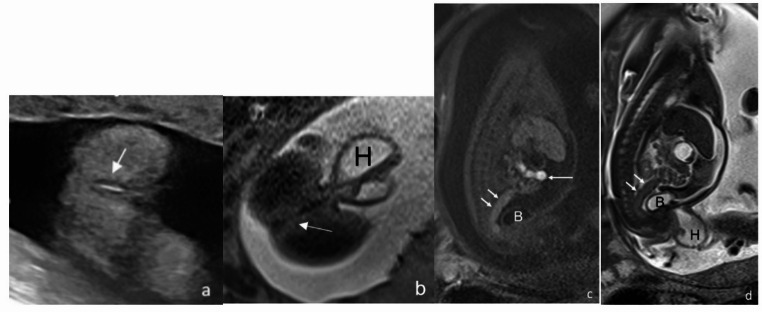
Imaging findings of anorectal malformation. Axial US (**a**) and T2-W MR (**b**) images of the fetal perineum at 28GW show absent anal “target sign” (arrow). Midline sagittal T1-W (**c**) and T2-W (**d**) images reveal normal signal of the meconium in the transverse colon (arrow, **c**) but abnormal signal in the rectum (short arrows, **c** and **d**), suggesting recto-urinary communication, confirmed at birth. Urinary ascites and prominent hydrocele (H) were also noted in this fetus with associated renal anomalies (not shown) (B: Bladder)

Colonic atresia is commonly located proximal to the splenic flexure and accounts for about 5–15% of bowel atresia. Association with other congenital anomalies, mostly also gastrointestinal, is observed in approximately 30% of cases. It is difficult to identify prenatally, as the amniotic fluid index often remains normal, and proximal small bowel dilatation may be absent [18]. Anorectal malformation (ARM) is a complex spectrum of abnormalities, from imperforate anus to persistent cloaca. It can appear as an isolated congenital anomaly, but it is most often associated with other malformations, which are detected in approximately 70% of cases. They include VACTERL association, caudal regression syndrome and chromosomal abnormalities [34, 35]. The prenatal detection rate of anal atresia remains low - about 16% - and the diagnosis in utero is difficult, especially when isolated [3, 34]. An absent perineal “target sign” is the most important direct sign, followed by a distended, thick walled and high-located rectum, but visualization of the “target sign” cannot completely exclude low anal atresia [36]. Abnormal echogenicity and signal intensity of the rectal meconium suggests a communication between the rectum and the genito-urinary tract with secondary mixture of fluids. The presence of a fistula reduces the incidence of polyhydramnios, which if present, can be due to other associated anomalies (Fig. 6) [36].

**Fig. 7:**
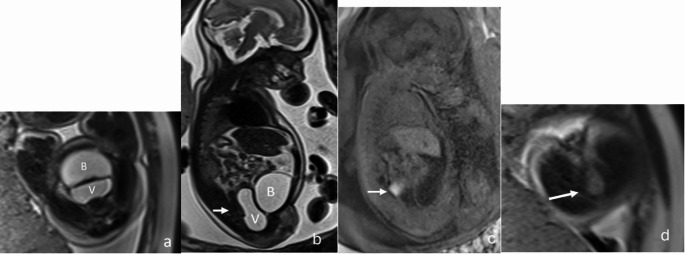
Imaging findings of confirmed cloaca. Transverse (**a**), sagittal T2-W (**b**) and sagittal T1-W (**c**) images of a female fetus reveal identical signal intensity of the fluids filling the bladder (B) and the distended vagina (V), suggesting communicating organs. Note the displaced position of the rectum (arrows, **b**, **c**). Perineal-oriented T2-W image (**d**) shows absent anal “target sign” (arrow)

Cloaca is the most severe form of ARM in females. In this pathology, the distal genital, urinary, and digestive tracts finish in a single common channel with a single external drainage [18]. Imaging findings include absent perineal “target sign”, a variable distended, fluid-filled vagina (hydrocolpos), and an often-displaced, high-located rectum with anomalous meconium signal. Homogenization of the fluids filling the different organs confirms their communication (Fig. 7) [2, 3, 29, 35].

In fetuses with atresia, MRI provides multiplanar imaging and allows differentiation between normal and abnormal meconium. These features help determine the level of obstruction, clarify complex anatomy, and identify associated congenital or multisystemic anomalies, particularly in syndromic cases [18]. MRI also improves the detection of fistulas [2, 3, 36].

### Multifocal digestive atresia

**Fig. 8:**
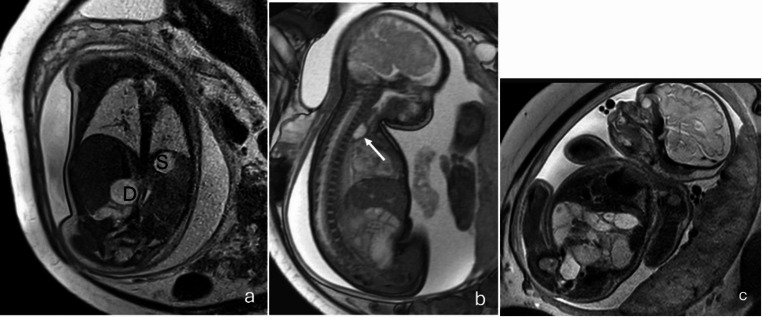
Multiple levels of atresia. In case 1 (**a**, **b**), MRI exam was performed at 30GW after US diagnosis of polyhydramnios and duodenal atresia. Coronal T2-W image (**a**) shows a classical “double-bubble sign” with a distended stomach (S) and duodenum (D). The midline sagittal T2-W image (**b**) reveals a “cervical pouch sign” (arrow), not previously detected. In case 2, a coronal T2-W MR image (**c**) at 34 GW shows distended intestinal loops filled with fluids with different signal intensities, indicating multiple levels of obstruction

Multiple levels of digestive tract obstruction increase the risk of fetal and neonatal complications, postoperative complications, and perinatal mortality [4]. The jejunum is the most frequent site of multiple-level bowel atresia. Multifocal obstructions modify the typical imaging findings previously described. Sonographic real-time evaluation may identify hyperperistalsis of the distended bowel and changes in the gain help to differentiate between the fluids filling the intestinal loops [31, 32]. However, a complete prenatal diagnosis on US remains challenging and limits an accurate diagnosis of the levels of obstruction despite technical advances. MRI can show different signal intensities on T1- and T2-W sequences of the fluids located at different levels, with low T1- bowel fluid evident in cases of upper jejunal atresia and more intermediate T1- and T2- W signal in distally located obstructions [4] (Fig. 8). MRI can confirm a suspected ultrasonographic diagnosis, increase the detection rate for concomitant atresia, and help to identify the levels of obstruction [4, 14, 37].

### Enteric duplication cysts

Duplication cysts are benign lesions that result from abnormal gastrointestinal tract recanalization, with a reported incidence of approximately 1:4500-10.000 births [38]. Associated digestive anomalies are observed in approximately 10% of cases and non-digestive in approximately 16–26% [39]. Duplication cysts can develop at any point along the digestive system, with the ileum being the most frequently observed location (33%), followed by the esophagus (20%) and colon (13%) [32]. Histological examination confirms the diagnosis by identifying a cyst that shares a common wall with the gastrointestinal tract, contains a of smooth muscle layer, and has an internal epithelial lining [38].

**Fig. 9:**
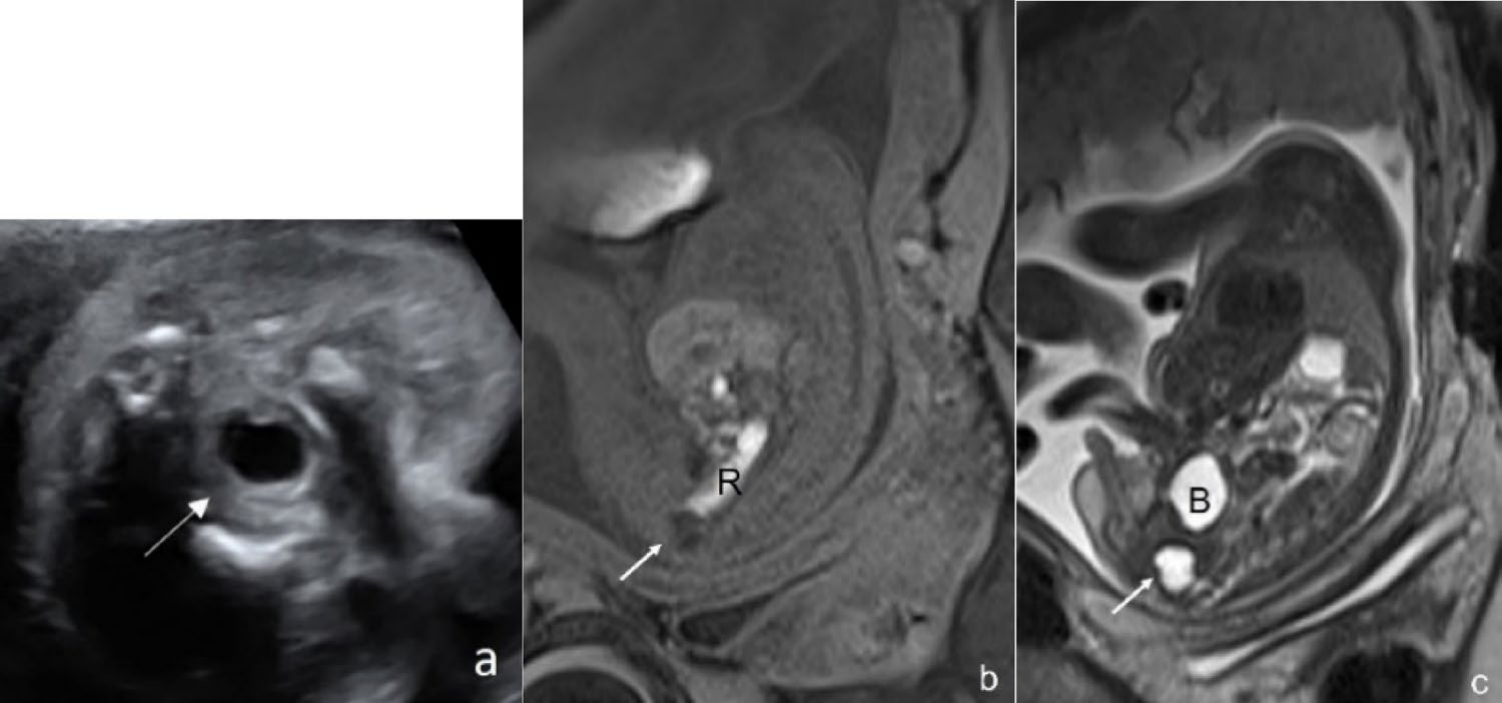
Imaging findings in rectal duplication cyst. Axial US image (**a**) at 25GW shows a pre-sacral located, well-defined cystic lesion in the fetal pelvis (arrow), with a thick wall, but provides limited value in evaluation of the adjacent organs. Midline sagittal T1- (**b**) and T2-WI (**c**) images of MRI at 30GW confirm the thick wall of the cyst (arrows) and show its direct anatomical relationship with the rectum (R), filled with normal meconium, (B: Bladder). The absence of contact with the coccyx excludes sacrococcygeal teratoma

Prenatal US identifies the pathology in only about 20–30% of cases [40]. US examination typically features an elongated, tubular or spherical, mostly unilocular cyst, in direct contact with the digestive wall, and with a thick and well-defined multilayered wall [41]. The classic “double wall sign” is considered the most important finding for distinguishing duplication cysts from other more frequently observed fetal cysts (such as the ovarian cyst in females), but it is not always present on US [42]. MRI may reveal the direct anatomical relationship between the cyst and the gastrointestinal tract, which helps to confirm the diagnosis in inconclusive cases (Fig. 9) [41, 43]. Communication with the intestinal lumen remains difficult to clarify *in utero* but, in our experience, fluid with meconium-like signal intensity characteristics filling colonic or rectal located cysts suggests communication.

## Gastrointestinal complications *in utero*

### Meconium peritonitis

**Fig. 10:**
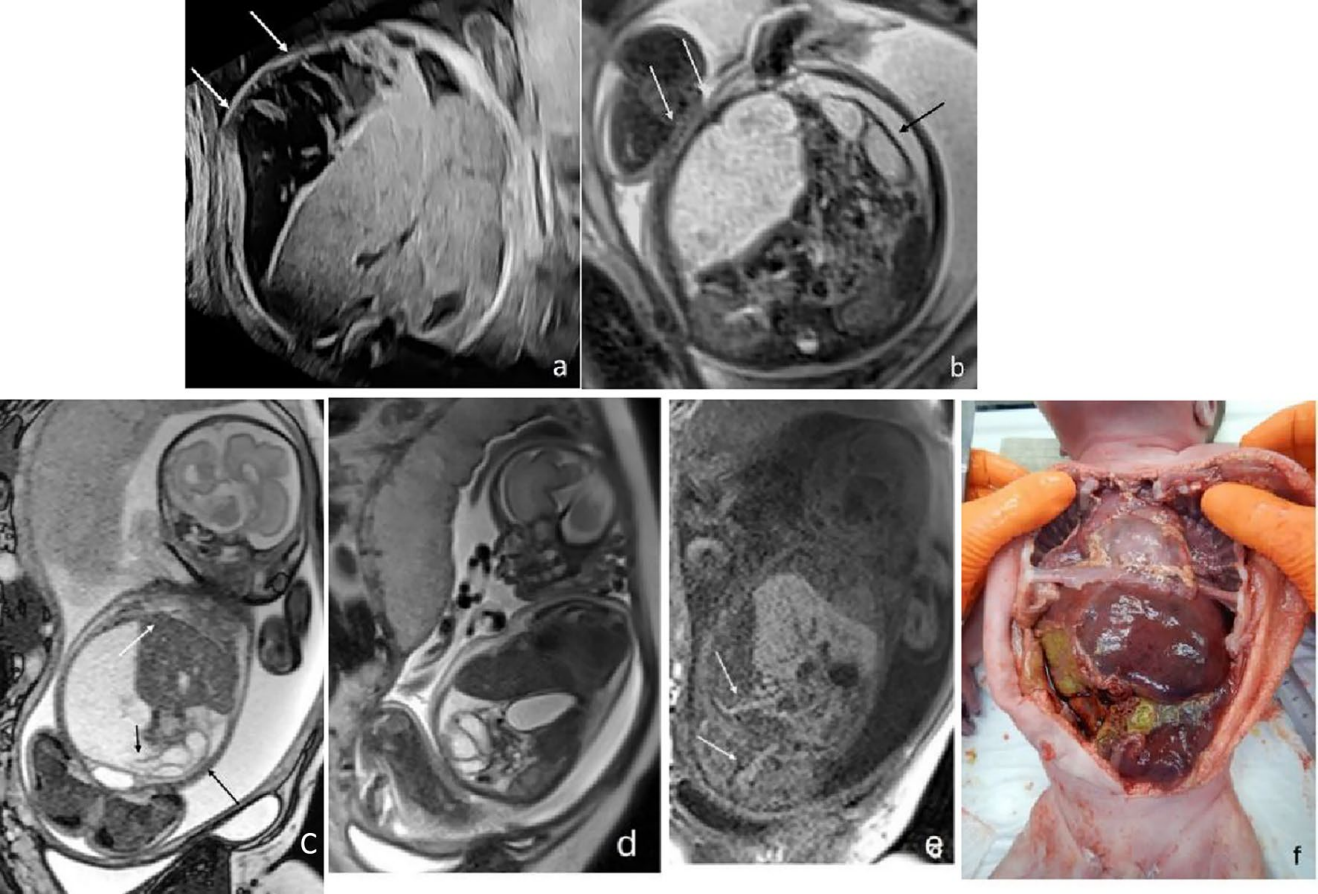
Meconium peritonitis following intestinal volvulus and perforation. Axial US of the upper abdomen (**a**) and axial (**b**), coronal (**c**), and sagittal (**d**) T2-W MR images at 30GW show voluminous ascites with calcifications and multiple septations (white arrows, **b**) causing significant hepatic distortion. Note multiple distended, fluid-filled bowel loops in the left hemiabdomen (black arrows, **b**, **c**), with suspected bowel perforation (**c**, short black arrow). Coronal T1-W image (**e**) reveals microcolon and rectum (white arrows, **e**), reduced meconium volume, and anomalous meconium signal for gestational age. Note also diaphragmatic elevation and severe bilateral lung hypoplasia (white arrow, **c**). Autopsy after *in utero* fetal demise revealed a conglomerate of necrotic intestinal loops, entangled and encased in a fibrous meconium plastron secondary to volvulus and perforation (**f**)

Meconium peritonitis has a reported incidence of approximately 1:35000 live births [8]. It results from intestinal perforation *in utero*, due to a wide spectrum of pathologies, including fetal infections, intestinal atresia, or bowel ischemia [44]. Leakage of meconium in the peritoneal cavity after perforation produces aseptic chemical peritonitis with an intense fibroblastic reaction [8]. Typical imaging findings include variable bowel dilatation, fetal ascites with septations and peritoneal calcifications, and meconium-filled pseudocyst formation [45]. US is better than MRI for detecting peritoneal calcifications, but the altered fetal anatomy and a limited field of view complicate the accurate identification of the level and the cause of intestinal pathology. MRI can provide a more detailed characterization of the anomalous digestive tract based on the location of the dilated loops, the colon’s morphology, and the distribution, quantity, and signal intensity of meconium [4]. This method can occasionally detect the level of perforation and help to detect and evaluate severity of associated complications (Fig. 10).

### Midgut and intestinal volvulus

In midgut volvulus, the intestinal loops are twisted around the superior mesenteric artery’s pedicle axis. The pathology is rare in fetuses, and its prenatal diagnosis remains challenging because the “whirlpool sign”, which shows the intestinal loops winding around the artery, is not always visible *in utero* [45, 46]. Another important sign is the “coffee bean sign” in which the distended closed loop of the intestine presents an elliptical shape, and the thickened intestinal walls converge in a central dense line (Fig. 5) [46]. Prenatal diagnosis of midgut volvulus does not necessarily require immediate intervention in fetuses with stable clinical status and dilated bowel, but requires close fetal monitoring and regular follow-up examinations. Significant reduction in active fetal movements, detection of absent peristalsis or intestinal hemorrhage, and development of signs of perforation and peritonitis are indications for urgent delivery and are associated with high rates of fetal and neonatal death (Fig. 5) [47, 48].

Segmental intestinal volvulus can occur following infection, atresia, or bowel ischemia. Indirect imaging findings include polyhydramnios, ascites, dilated intestinal loops, and meconium peritonitis with pseudocysts after perforation (Fig. 10). In these cases, MRI can help to identify the cause and location of the pathology, and to evaluate associated complications (Figs. 5 and 10) [47, 48].

## Conclusions

Prenatal US screening has greatly improved the detection of fetal gastrointestinal pathologies and complications. In selected cases with inconclusive ultrasonographic diagnosis, MRI may facilitate the identification of the level of obstruction and increase the detection rate of concomitant obstructions in fetuses with atresia, detect unexpected associated anomalies in patients with certain syndromes and multisystemic pathologies, and help to identify the cause and location of digestive complications *in utero*. The additional information obtained at MRI improves diagnosis, contributes to better evaluation of prognosis during pregnancy and after birth, and helps clinicians with management of complicated pregnancies.

## Data Availability

No datasets were generated or analysed during the current study.

## Abbreviations

ARM: Anorectal malformation
EUROCAT: European registry of congenital anomalies
GW: Gestational weeks
MRI: Magnetic resonance imaging
EA: Esophageal atresia
TEF: Tracheo-esophageal fistula
US: Ultrasound
